# Evaluating the Role of the Interleukin-23/17 Axis in Critically Ill COVID-19 Patients

**DOI:** 10.3390/jpm11090891

**Published:** 2021-09-07

**Authors:** Edison Jahaj, Alice G. Vassiliou, Chrysi Keskinidou, Parisis Gallos, Charikleia S. Vrettou, Stamatios Tsipilis, Zafeiria Mastora, Stylianos E. Orfanos, Ioanna Dimopoulou, Anastasia Kotanidou

**Affiliations:** 1 First Department of Critical Care Medicine & Pulmonary Services, School of Medicine, National & Kapodistrian University of Athens, Evangelismos Hospital, 106 76 Athens, Greece; Edison.jahaj@gmail.com (E.J.); alvass75@gmail.com (A.G.V.); chrysakes29@gmail.com (C.K.); vrettou@hotmail.com (C.S.V.); stamostsipil@gmail.com (S.T.); zafimast@yahoo.gr (Z.M.); sorfanos@med.uoa.gr (S.E.O.); idimo@otenet.gr (I.D.); 2 Computational Biomedicine Laboratory, Department of Digital Systems, University of Piraeus, 185 34 Piraeus, Greece; parisgallos@yahoo.com

**Keywords:** COVID-19, IL-17, IL-23, critically ill, personalized treatment

## Abstract

Studies have hypothesized a potential role of the interleukin (IL)-23/17 axis in coronavirus disease 2019 (COVID-19). However, to date, levels of IL-23 and 17 have not been compared between critically ill COVID-19 patients and critically ill non-COVID-19 patients. IL-23 and 17 were measured on admission to the intensive care unit (ICU) in critically ill COVID-19 (*N* = 38) and critically ill non-COVID-19 (*N* = 34) patients with an equal critical illness severity. Critically ill non-COVID-19 patients did not have sepsis or septic shock on ICU admission. None of the enrolled patients had previously received corticosteroids. In our study, circulating IL-17 levels were higher in the COVID-19 patients. More specifically, critically ill COVID-19 patients had levels of 0.78 (0.05–1.8) pg/mL compared to 0.11 (0.05–0.9) pg/mL in the critically ill non-COVID-19 patients (*p* = 0.04). In contrast, IL-23 levels were comparable between groups. A group of patients hospitalized in the specialized COVID-19 clinic (*N* = 16) was also used to evaluate IL-17 and IL-23 levels with respect to COVID-19 severity. Non-critically ill COVID-19 patients had undetectable levels of both cytokines. Our results support the notion of inhibiting IL-17 in critical COVID-19 infection.

## 1. Introduction

Severe acute respiratory syndrome coronavirus 2 (SARS-CoV-2) infection exhibits a wide diversity of symptoms, resulting in a plethora of clinical manifestations, and variable disease severity. Patients with moderate to severe disease develop lung injury, most commonly coronavirus disease 2019 (COVID-19) pneumonia [1]. A common complication of more severe cases is the development of acute respiratory distress syndrome (ARDS) [2]. It has been suggested that the diversity in symptom severity, and eventually the patients’ adverse outcome, is most likely attributed to differences in the host’s immunological profile, rather than virus variation [3]. Viral infection is often accompanied by an excessive inflammatory response, termed “cytokine storm”, which damages the endothelial integrity and facilitates the induction of endothelitis [4]. The immunopathology of COVID-19 is currently at the center of basic and clinical research. Multiple studies have reported highly elevated levels of pro-inflammatory cytokines in COVID-19 and have linked the cytokine storm with severe complications and poor outcomes. However, there is currently a limited understanding of the cytokine storm in severe COVID-19.

The interleukin (IL)-23/17 axis plays a central role in the development of inflammation, and in host defense against bacterial infection [5]. The IL-23/17 axis has been associated with inflammation, as well as a variety of diseases and autoimmunity. IL-23 induces the differentiation of naive cluster of differentiation 4^+^ (CD4^+^) T cells into IL-17-producing T-helper cells (Th17) [5]. IL-17 is one of the many cytokines released during COVID-19 [6]. IL-17 is a pro-inflammatory cytokine, mainly produced by Th17 cells, but also by innate and other adaptive immune cells; it is crucial in recruiting and activating neutrophils, which are heavily involved in the pathogenesis of COVID-19 [7]. Neutralizing IL-23 or IL-17 therapeutic strategies appear to have a good impact on immune-mediated inflammatory disorders such as psoriasis, multiple sclerosis, and rheumatoid arthritis (RA) [8]. The study of the involvement of the IL-23/17 axis in COVID-19 could have significant implications. A presumed central role of this axis to the pathophysiology of COVID-19-related ARDS could suggest the inhibition of this pathway as a therapeutic strategy. The several IL-17 antagonists that are used in the treatment of autoimmune conditions could also be potential beneficial treatments for severe COVID-19 cases [7].

In a previous study, we concluded that the characterization of the cytokine storm seen in COVID-19 depends on control group selection [9]. To the best of our knowledge, however, no study has measured IL-23 and IL-17 levels in critically ill COVID-19 and non-COVID-19 patients. To this end, we aimed to compare IL-23 and IL-17 levels between critically ill COVID-19 patients and critically ill non-COVID-19 patients with an equal disease severity. All of the patients studied were steroid-free. Moreover, non-COVID-19 patients did not have sepsis or septic shock on intensive care unit (ICU) admission. An additional group of patients hospitalized in the specialized COVID-19 clinic was also used for comparison.

## 2. Materials and Methods

This observational study was conducted in one general ICU and one specialized COVID-19 clinic. Our prospective study adhered to the ethical standards of the research committee, and the current Helsinki Declaration. The study received approval (129/19 March 2020) from the Hospital Research Ethics Committee. All patients included in the study or the patients’ next-of-kin provided us with their informed consent. ICU or ward admission (within 24 h) serum concentrations of IL-23 (detection limit: 16.3 pg/mL) and IL-17 (detection limit: 0.051 pg/mL) were concurrently determined by the enzyme-linked immunosorbent assays (ELISA) (R&D Systems, Inc, Minneapolis, MN, USA), by the same researcher, in 38 consecutive critically ill COVID-19 patients, 34 consecutive critically ill non-COVID-19 patients, and 16 non-critically ill COVID-19 patients hospitalized in the specialized COVID-19 clinic of our hospital. All samples were assayed in duplicate. COVID-19 infection was confirmed by real-time reverse transcription PCR (RT-PCR) in nasopharyngeal swabs. All of the critically ill COVID-19 patients included in our study presented with pneumonia and were directly admitted to the ICU from March 22 to 5 October 2020. The non-critically ill patients were hospitalized in the specialized COVID-19 clinic of our hospital from 11 April to 14 October 2020. Dexamethasone administration and age < 18 years constituted the exclusion criteria. The critically ill non-COVID-19 patients were part of a previously studied cohort [10].

Regarding the statistical analyses, descriptive statistics were calculated, and two-group comparisons were performed using the Student’s *t*-test or the Mann–Whitney test for the non-parametric variables. In addition, the chi-square test for qualitative variables and Spearman’s correlation coefficient for quantitative variables were calculated. For descriptive statistics, mean ± standard deviation (SD) for normally distributed variables, median with interquartile range (IQR) for variables with skewed distribution, and count (%) when categorical, were also calculated using the GraphPad software (GraphPad Software, Inc., San Diego, CA, USA). For all the bivariable analyses, a *p*-value < 0.05 was considered statistically significant.

## 3. Results

**Figure 1:**
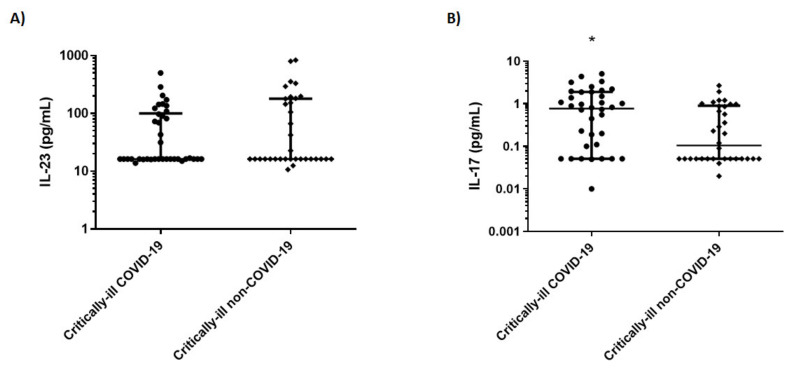
Interleukin levels in critically ill COVID-19 and non-COVID-19 patients. IL-23 (**A**) and IL-17 (**B**) were measured on ICU admission in 38 COVID-19 patients and 34 non-COVID-19 critically ill patients. Data are presented as scatter plots showing median and interquartile range. Line in the middle, median value; upper and lower lines, 25th to 75th centiles. * *p* < 0.05 by Mann–Whitney. COVID-19 = coronavirus disease 19; IL: interleukin.

The critically ill non-COVID-19 patients consisted of trauma patients (32.4%), surgical patients (32.4%), and medical (central nervous system, CNS-related pathologies) cases (35.2%). The two critically ill groups did not differ in terms of disease severity (Table 1). COVID-19 patients manifested the first symptoms 6 ± 2 days prior to ICU admission. COVID-19-inflicted ARDS [11] was present in 90% of the patients. As has been documented, the critically ill COVID-19 patients were older, mostly male, and had more comorbidities compared to the population of a general ICU. In our study, circulating IL-17 levels were higher in critically ill COVID-19 patients compared to non-COVID-19 critically ill patients (Table 1 and Figure 1). The subset of COVID-19 inflicted ARDS patients (*N* = 34) also had higher IL-17 levels compared to the non-COVID-19 patients suffering from ARDS (*N* = 20) (0.86 vs. 0.24, *p* < 0.05). Among the critically ill COVID-19 patients, ICU admission IL-17 levels did not differ between survivors and non-survivors (0.73 pg/mL vs. 0.69 pg/mL, *p* > 0.05), but tended to be higher in patients with COVID-19-inflicted ARDS requiring invasive mechanical ventilation compared to those who were not intubated (0.86 pg/mL vs. 0.08 pg/mL, *p* = 0.08). IL-23 levels were comparable between the two critically ill cohorts (Table 1 and Figure 1). In the non-critically ill COVID-19 patients, the levels of both interleukins were undetectable (Table 1).

**Figure 2:**
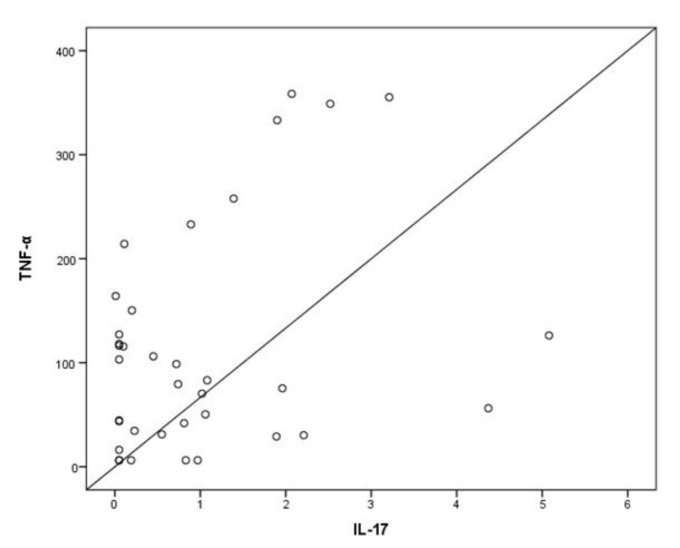
Spearman’s correlation coefficient analysis was performed on IL-17 and TNF-α levels evaluated on ICU admission (within 24 h). IL-17: interleukin-17; TNF-α: tumor necrosis factor-α.

In the critically ill COVID-19 patients, levels of IL-17 and IL-23 did not exhibit a correlation (*p* > 0.05). Using previously published data of the critically ill COVID-19 patients [9], we found that IL-17 levels positively correlated with tumor necrosis factor α (TNF-α) levels (r_s_ = 0.33, *p* = 0.049; Figure 2). IL-17 did not correlate with the other previously measured interleukins (IL-6, IL-8, and IL-10), while IL-23 did not correlate with any of the cytokines previously measured.

## 4. Discussion

To the best of our knowledge, no other study has measured IL-23 and IL-17 levels in critically ill COVID-19 patients, or compared their levels to critically ill non-COVID-19 patients and non-critically ill COVID-19 patients. In the present study, we found that IL-17 levels in critically ill COVID-19 patients were higher compared to critically ill non-COVID-19 subjects, with an equal critical illness severity. Moreover, IL-17 levels were undetectable in non-critically ill COVID-19 patients. These findings might support a potential role of IL-17 inhibition in critical COVID-19 infection.

Despite a potential role of IL-17 as an intervention target for COVID-19, clinical data are currently limited. In two previous studies, COVID-19 patients were found to have higher IL-17 levels compared to healthy subjects [12,13]. In another study, no significant differences were found in IL-17 between severe and non-severe COVID-19 cases [14], while no difference was detected in COVID-19 patients and healthy subjects [15]. Many reports have also suggested a potential role of IL-17 as a target in COVID-19. IL-17 has been linked to viral load in a small number of COVID-19 patients, and the authors proposed its use as a potential biomarker of COVID-19 severity [16]. In our critically ill COVID-19 patients, ICU admission IL-17 levels did not differ in survivors and non-survivors; they tended, however, to be higher in patients with COVID-19-inflicted ARDS requiring invasive mechanical ventilation compared to non-ARDS patients. Moreover, IL-17 positively correlated with TNF-α levels in the critically ill COVID-19 patients. Previous results from our group have shown that circulating IL-6 and IL-10 were lower, while TNF-α and IL-8 levels were higher, in the same critically ill COVID-19 patients compared to the critically ill non-COVID-19 patients [9]. The synergistic action of IL-17 with TNF-α has been shown to have major pro-coagulant and pro-thrombotic effects [17,18], and furthermore, combined blockade of TNF-α and IL-17 has been shown to be a safe treatment strategy in autoimmune diseases where monotherapy is not fully effective [19].

Besides the main IL-17 producers, CD4^+^ and CD8^+^ cells, different subtypes of innate lymphocytes, such as gamma-delta (γδ) T cells, natural killer T cells (NKT), and group 3 innate lymphoid cells (ILC3), also contribute to IL-17 production [20,21]. During infection, NKT cells migrate from the circulation to the lung, where they form aggregates. An increased concentration of highly activated NKTs is observed, following chemokine release in the bronchoalveolar lavage (BAL) of patients with COVID-19 [22]. NKT cells are important antiviral effectors due to their ability to respond without a prior sensitization, and therefore, are able to produce IL-17 immediately following antigen exposure. In contrast, IL-17 production from CD4^+^ T cells is more complex, and depends on different factors and cytokines [6,21,22]. Likewise, γδ T cells have been characterized as a primary source of IL-17 secretion in several in vitro lung injury models [20]. It appears plausible that the innate IL-17-producing cells mentioned above, through their ability to express transcriptional IL-17 regulators, rapidly produce IL-17 within the first hours of antigen stimulation and, therefore, could have an integral role in the formation of the adaptive immune response [20]. Hence, it seems that Th17 plays a role in the pathogenesis of COVID-19 pneumonia, not only by inducing the cytokine cascade, but also by modulating the adaptive immune response [6].

Since the release of the RECOVERY trial results, dexamethasone has been recommended and applied as a principal treatment for COVID-19 hospitalized patients who require supplemental oxygen [23]. Daily administration of dexamethasone increased the 28-day survival in patients receiving respiratory support, when administered more than 7 days following symptom onset. At this phase, inflammatory lung damage is more likely. Dexamethasone is a glucocorticoid that has been used as a treatment in a wide range of diseases. Its quick anti-inflammatory and immunosuppressive properties render it a suitable candidate for controlling the immune-mediated lung damage present in severe COVID-19 cases [24]. Corticosteroids inhibit the production of pro-inflammatory cytokines, such as IL-17, repressing the associated COVID-19 hyper-inflammation [24,25]. In our cohort, the patients were from the “first wave” and had not received corticosteroids. It is possible that dexamethasone administration would have alleviated IL-17 production, and systemically promoted a more anti-inflammatory phenotype.

The inhibition of IL-23 and IL-17 with monoclonal antibodies is a very effective therapy for psoriasis, psoriatic arthritis, and other autoimmune diseases. Case reports have shown that patients with psoriasis treated with IL-17A antagonists, including secukinumab and ixekizumab, showed relatively mild disease or were asymptomatic when they were infected with SARS-CoV-2 [26,27,28,29]. Similar to psoriasis, imbalance of the pro- and anti-inflammatory cytokines is also seen in ARDS. The increased pro-inflammatory cytokine production in the lungs is characterized by diffuse alveolar damage, neutrophil recruitment, and protein-rich edema in the alveolar space [30]. In fact, in the influenza A virus subtype H1N1 pandemic, IL-17 blocking agents improved the response in mice, by reducing inflammatory cell recruitment to the lungs, cytokine production, and lung edema formation [31]. These wide favorable actions support a protective role of IL-17 inhibitors in COVID-19 [32]. Very recently, in a pilot case-control study, it was demonstrated that the patients with severe COVID-19 treated with anti-IL-17 monoclonal antibodies showed moderated inflammatory response and improved oxygenation; however, treatment did not seem to improve survival, or reduce mechanical ventilation requirement [33]. With the aim of achieving new target-specific drug therapies, examining cytokines known to be associated with chronic hyper-inflammatory diseases could potentially lead us in repurposing drugs and improving the existing therapeutic strategies.

The limitations of our study must be mentioned. This was a single-center study with a limited sample size, not allowing for generalization. Multi-center studies with a larger patient population are needed to confirm our findings. The strengths include the fact that the two groups of critically ill patients had an equal disease severity. Another strength is the fact that we also included non-critically ill COVID-19 patients.

The results of our study are important for at least two reasons. One is that measuring serum IL-17 levels could provide a biomarker for the evolution of lung involvement in COVID 19. Studies with serial measurements are needed in order to support this hypothesis. Another is that the plethora of monoclonal antibodies that can inhibit the Th17 response, and are currently used in autoimmune conditions, could potentially be beneficial in treating COVID-19-induced ARDS. Measuring the levels of specific cytokines could possibly help us to identify those COVID-19 patients who could benefit the most from monoclonal antibody therapies. This personalized treatment option could provide us with improved outcome status.

## 5. Conclusions

In our cohort, IL-17 levels in critically ill COVID-19 patients were higher compared to critically ill non-COVID-19 subjects, with an equal critical illness severity. Given our results, and the so far produced clinical trial reports in COVID-19 patients, more attention needs to be brought to the IL-23/17 axis. A deeper comprehension of the IL-23/17 axis could help design new and more personalized therapies.

## Figures and Tables

**Table 1:** Patient characteristics on admission.

Parameters	Critically Ill	Critically Ill	Non-Critically Ill
	COVID-19	Non-COVID-19	COVID-19
	(*N* = 38)	(*N* = 34)	(*N* = 16)
Age, mean ± SD, years	63 ± 11	50 ± 21 **	55 ± 15 *
Sex, N (%)			
Male	31 (81.6)	20 (58.8) *	8 (50.0) *
Female	7 (18.4)	14 (41.2)	8 (50.0)
Comorbidities, N (%)	25 (65.8)	10 (29.4) **	9 (56.3)
Diabetes	5	3	2
Hypertension	17	5	2
CAD	4	1	-
COPD	1	1	-
ARDS, N (%)	34 (89.5)	20 (58.8) **	4 (25.0) ****
PaO_2_/FiO_2_, mean ± SD, mmHg	196 ± 86	280 ± 140 **	342 ± 90 ****
APACHE II score, mean ± SD	15 ± 6	12 ± 4	NA
SOFA score, mean ± SD	7 ± 3	6 ± 2	NA
CRP, median (IQR), mg/dL	11.8 (5.3–19.8)	4.1 (1.8–10.9) **	2.6 (0.6–11.8) **
White blood cell count, mean ± SD, cells/µL	10220 ± 4700	10830 ± 3350	6070 ± 3670 **
IL-23, median (IQR), pg/mL	16.3 (16.3–100.5)	16.3 (16.3–180.4)	Undetectable
IL-17, median (IQR), pg/mL	0.78 (0.05–1.89)	0.11 (0.05–0.91) *	Undetectable
Mortality, N (%)	10 (27.8)	5 (16.7)	1 (6.3)

Data are expressed as number of patients (N) and percentages of totals (%), mean ± SD, or median (IQR), as appropriate. Two-group comparisons were performed against the critically ill COVID-19 patients using either the Student’s *t*-test or the non-parametric Mann–Whitney test, as appropriate. The chi-square test was used to assess associations between qualitative variables. All parameters were estimated within the first 24 h post-admission. IL-23 levels were undetectable in 17 (44.7%) critically ill COVID-19 patients and 17 (50.0%) critically ill non-COVID-19 patients, whereas IL-17 levels were undetectable in 7 (18.4%) critically ill COVID-19 patients and 13 (38.2%) critically ill non-COVID-19 patients. APACHE = acute physiology and chronic health evaluation; ARDS = acute respiratory distress syndrome; CAD = coronary artery disease; COPD = chronic obstructive pulmonary disease; COVID-19 = coronavirus disease 2019; CRP = C-reactive protein; IL = interleukin; IQR = interquartile range; NA = not applicable; SD = standard deviation; SOFA = sequential organ failure assessment. * *p* < 0.05; ** *p* < 0.01; *** *p* < 0.001; **** *p* < 0.0001 from the critically ill COVID-19 patients.

## Data Availability

Data supporting reported results are available upon reasonable request.
